# Novel avian influenza A (H5N6) viruses isolated in migratory waterfowl before the first human case reported in China, 2014

**DOI:** 10.1038/srep29888

**Published:** 2016-07-19

**Authors:** Yuhai Bi, Haizhou Liu, Chaochao Xiong, Di Liu, Weifeng Shi, Mingxin Li, Siling Liu, Jing Chen, Guang Chen, Yong Li, Guoxiang Yang, Yongsong Lei, Yanping Xiong, Fumin Lei, Hanzhong Wang, Quanjiao Chen, Jianjun Chen, George F. Gao

**Affiliations:** 1 Shenzhen Key Laboratory of Pathogen and Immunity, State Key Discipline of Infectious Disease, Shenzhen Third People’s Hospital, Shenzhen 518112, China; 2 Key Laboratory of Special Pathogens and Biosafety, Wuhan Institute of Virology, Chinese Academy of Sciences, Wuhan 430071, China; 3 Key Laboratory of Pathogenic Microbiology and Immunology, Institute of Microbiology, Chinese Academy of Sciences, Beijing 100101, China; 4 Center for Influenza Research and Early-warning (CASCIRE), Chinese Academy of Sciences, Beijing 100101, China; 5 Institute of Pathogen Biology, Taishan Medical College, Taian 271016, China; 6 The Monitoring Center of Wildlife Diseases and Resource of Hubei Province, Wuhan 430075, China; 7 CAS Key Laboratory of Zoological Systematics and Evolution, Institute of Zoology, Chinese Academy of Sciences, Beijing 100101, China; 8 Office of Director-General, Chinese Center for Disease Control and Prevention (China CDC), Beijing 102206, China; 9 University of Chinese Academy of Sciences Medical School, Chinese Academy of Sciences, Beijing 101408, China

## Abstract

In May 2014, China formally confirmed the first human infection with the novel H5N6 avian influenza virus (AIV) in Sichuan Province. Before the first human case was reported, surveillance of AIVs in wild birds resulted in the detection of three H5N6 viruses in faecal samples from migratory waterfowl in Chenhu wetlands, Hubei Province, China. Genetic and phylogenetic analyses revealed that these three novel viruses were closely related to the H5N6 virus that has caused human infections in China since 2014. A Bayesian phylogenetic reconstruction of all eight segments suggests multiple reassortment events in the evolution of these viruses. The hemagglutinin (HA) and neuraminidase (NA) originated from the H5N2 and H6N6 AIVs, respectively, whereas all six internal genes were derived from avian H5N1 viruses. The reassortant may have occurred in eastern China during 2012–2013. A phylogeographic analysis of the HA and NA genes traced the viruses to southern China, from where they spread to other areas via eastern China. A receptor-binding test showed that H5N6 viruses from migratory waterfowl had human-type receptor-binding activity, suggesting a potential for transmission to humans. These data suggest that migratory waterfowl may play a role in the dissemination of novel H5N6 viruses.

Wild birds are known to play a major role in the evolution, maintenance, and spread of the avian influenza viruses (AIVs). Wild birds, especially waterfowl such as the Anseriformes (e.g., ducks, geese, and swans) and Charadriiformes (e.g., gulls, terns, and shorebirds), are thought to be the natural reservoirs of AIVs^1^,^2^. Almost all subtypes of avian influenza A viruses, H1–H16 and N1–N9, have been identified in wild birds^3^. Typically, wild birds infected with low-pathogenic avian influenza A viruses are asymptomatic or display subclinical symptoms^4^, allowing the virus to be spread over long distances with host migration. During infection, AIVs preferentially localize in the intestinal tracts of migratory waterfowl and are excreted in high concentrations in their faeces^5^,^6^. Therefore, transmission between wild waterfowl or between wild waterfowl and domestic poultry is thought to occur via the faecal–oral transmission route or by the ingestion of contaminated water^7^,^8^.

Wild birds are thought to play a role in the unexpected appearance of novel influenza A viruses in poultry, swine, equines, and humans^2^. The human pandemic strains that emerged in the last century contain genetic segments derived from AIVs of wild bird origin^9^. A novel reassortant human-infecting avian influenza A (H7N9) virus, first identified in China in March 2013, contains the neuraminidase (NA) gene from wild ducks in South Korea^10^. After the outbreak of H7N9, fatal human infections with a novel H10N8 virus were reported in China^11^, and the H10 gene was shown to have been introduced to domestic ducks by migratory ducks on Poyang Lake in multiple events^12^. Therefore, surveillance of circulating AIVs in wild birds is important in understanding the evolution and emergence of potentially pandemic strains.

Among the 16 HA subtypes in wild birds, the H5 and H7 viruses are thought to potentially convert to highly pathogenic (HP) strains by adaptation in poultry. Highly pathogenic avian influenza (HPAI) is generally considered to cause severe morbidity and mortality in poultry populations, and the ongoing circulation of HPAI H5 viruses in Southeast Asia since 1997 has had a devastating impact on the poultry industry in that area^13^. Before 2005, HPAI H5N1 viruses were only isolated sporadically from wild birds^14^, but an outbreak in Qinghai Lake during 2005 resulted in the deaths of many migratory birds^15^,^16^. Migratory birds infected with HPAIV may not pose a direct threat to public health, but may spread the virus across countries and continents via long-distance migration before transmission back to poultry populations^17^.

On May 6, 2014, a fatal human H5N6 infection was reported in Sichuan Province, China (China CDC, 2014; WHO, 2014b). The patient was a poultry dealer working in a live poultry market, which have been shown to play a key role in human infections with influenza A (H7N9 and H10N8) viruses^18^,^19^,^20^. Surveillance of AIVs in live poultry markets demonstrated that they are the source of H5N6 human infections with^21^. However, the role of wild birds in the spread of H5N6 is unclear. Here, we report the isolation and characterization of three previously unrecognized novel avian influenza A (H5N6) viruses in migratory waterfowl before the first human infection was reported.

## Results

### Isolation of H5N6 viruses from fresh faeces of migratory waterfowl

After the emergence of the H7N9 influenza viruses during early 2013 in China, active surveillance of AIV in wild birds was undertaken in the Chenhu wetlands. During December 2013–March 2014, a total of 1240 fresh faeces samples from wild birds were collected (Table S1). The sampling sites were located approximately 740 km from the site of the first human H5N6 infection, in Sichuan Province. At the time of sampling, no sick or dead birds were observed in the sampling area. Two H5N6 viruses were initially isolated from samples collected on February 26, 2014, and were designated A/Migratory waterfowl/Hubei/Chenhu1306/2014 (CH1306) and A/Migratory waterfowl/Hubei/Chenhu1347/2014 (CH1347), and their hosts were not identified. The third H5N6 virus was isolated from a sample collected on March 20, 2014, and designated A/Anas crecca/Hubei/Chenhu1623-5/2014 (CH1623-5).

### Origin of migratory waterfowl H5N6 viruses

**Figure 1:**
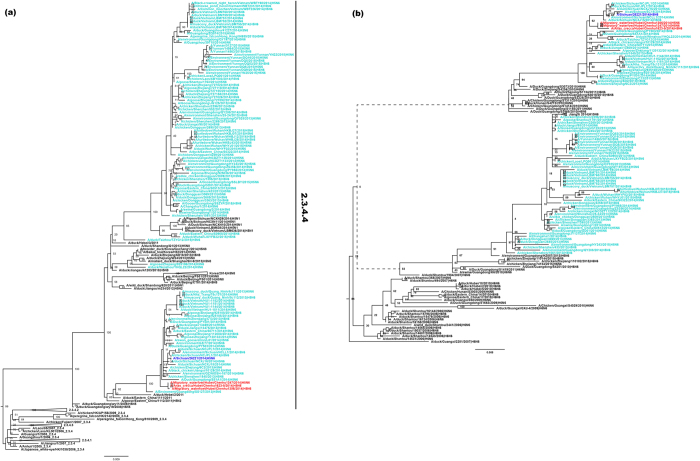
Maximum likelihood phylogenetic trees of HA and NA segments. (**a**) ML phylogenetic tree of HA segment, (**b**) ML phylogenetic tree of NA segment. Migratory birds H5N6 viruses reported in this paper are marked in red, and the human-originating strain A/Sichuan/26221/2014 is marked in blue. Bootstrap values less than 50% are not shown.

The whole-genome sequences of the three H5N6 influenza viruses were determined. To clarify the origins of the H5N6 viruses, we downloaded all available sequences (until May 2016) of novel H5N6 viruses, representative H5 strains, and other N6 viruses from the GenBank and GISAID databases. The phylogenetic tree constructed based on the HA segment suggested that these three strains were evolutionarily close to the H5N6 viruses isolated from poultry and environmental samples in China during 2013 and 2014 (Fig. 1a), and belonging to a new H5 clade, designated 2.3.4.4^22^. The phylogenetic tree of the NA segment confirmed the relationships between these three strains and other recently isolated H5N6 strains (Fig. 1b).

**Figure 2:**
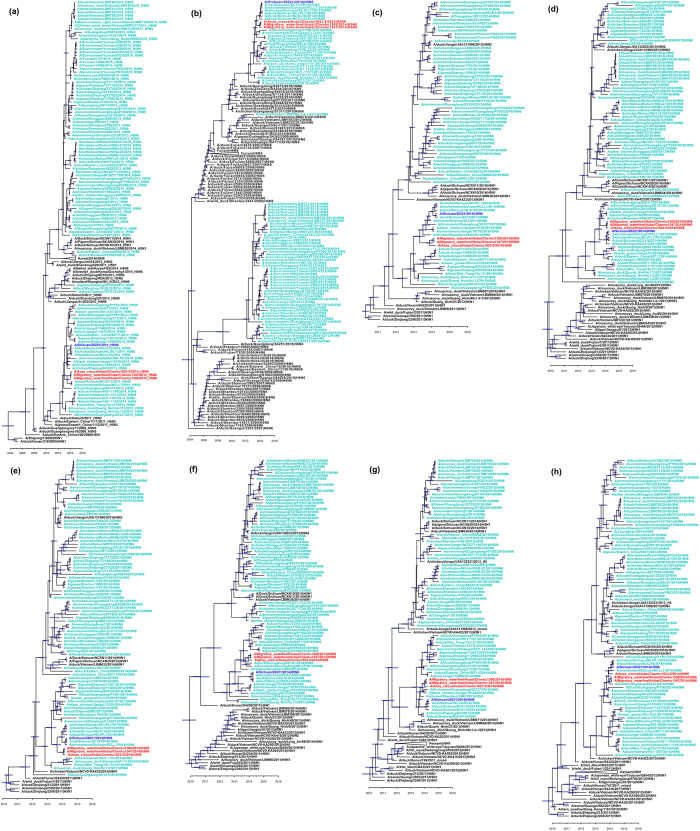
Maximum clade credibility trees of eight segments. (**a**) HA segment, (**b**) NA segment, (**c**) PB2 segment, (**d**) PB1 segment, (**e**) PA segment, (**f**) NP segment, (**g**) M segment, and (**h**) NS segment. Horizontal bar indicates the 95% HPD of each node. Strains in parentheses indicate that these strains share identical sequences with the sequence on the tree.

The most recent common ancestor (TMRCA) and more phylogenetic details of all the viral segments were inferred with maximum clade credibility (MCC) trees generated with BEAST and Tree Annotator software (Fig. 2). The MCC trees revealed that all eight segments of these three wild-bird-borne viruses were phylogenetically close to the human-origin strain A/Sichuan/26221/2014 (H5N6). According to the HA and NA MCC trees, these H5N6 lineages were composed of viruses isolated from poultry, wild birds, and environmental samples in 2013 and 2014 in China and Vietnam. Furthermore, this clade separated from its most recent HA ancestor (A/duck/Hebei/2/2011(H5N2)) in July 2010 and NA ancestor (A/duck/Guangdong/S3073/2010(H6N6)) in May 2010, suggesting a relatively long and unclear evolution period of these viruses. Besides this H5N6 viral lineage in waterfowl, there is another H5N6 viral lineage circulating at the same period, which consists of viruses isolated from poultry and the environment in China during 2013–2015, as well as strains from Vietnam and Laos in 2014. Both H5N6 lineages originated from the common HA ancestor (A/duck/Eastern China/108/2008(H5N1)) and NA ancestor (A/duck/Guangxi/2281/2007(H6N6)), but with independent evolution histories. However, the internal segments of both H5N6 lineages clustered into the H5N1 lineage, indicating that their internal segments share a similar evolutionary history.

**Figure 3:**
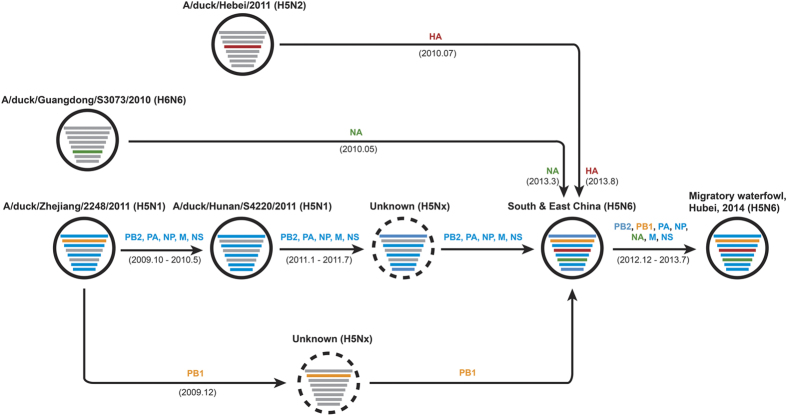
Hypothetical migration and evolutionary history of water fowl H5N6 AIVs.

Based on these eight MCC trees, a complicated evolutionary history, with multiple reassortments, can be inferred and is shown in Fig. 3. In this analysis, precursor H5N6 viruses circulated in South and East China during late 2012 and early 2013, in which HA, NA, and the internal gene segments originated from different ancestors. The HA segment was derived from an H5N2 virus and the NA segment was acquired from H6N6 strains persisting in the South and East China around 2010. With the exception of PB1, the other internal segments can be traced back to the A/duck/Zhejiang/2248/2011 (H5N1) strain.

### Phylogeography of the migratory waterfowl H5N6 viruses

**Figure 4:**
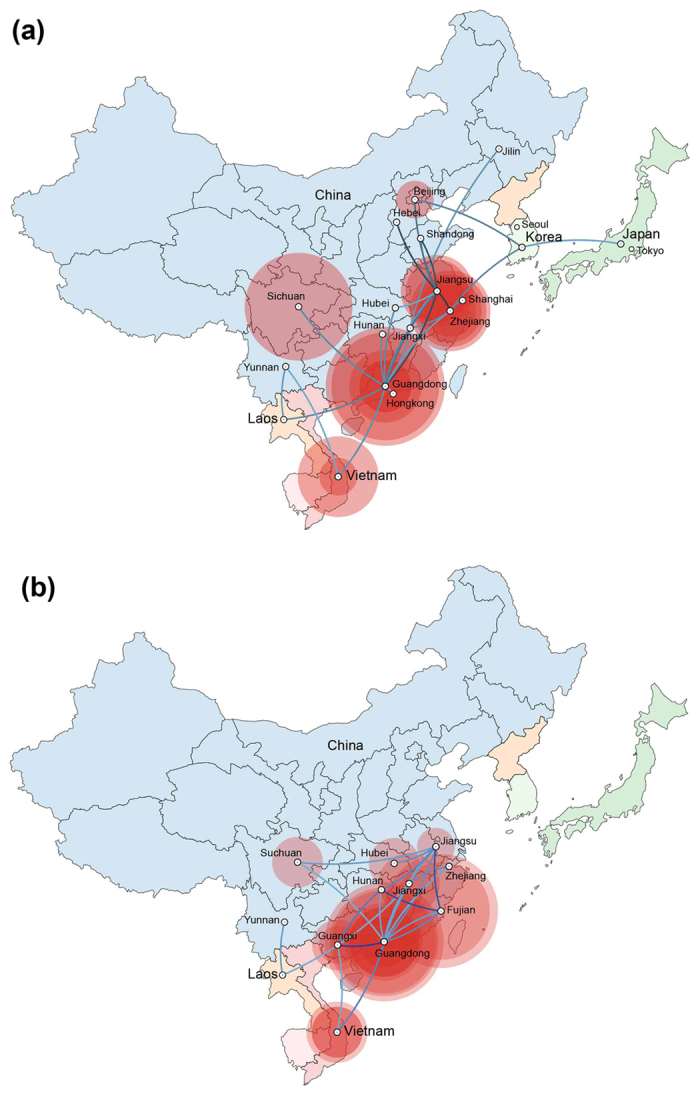
Dispersal patterns of HA (**a**) and NA (**b**) segments. Lines between locations represent branches on the MCC tree and the colour gradient indicated the height of location. Circle diameters of locations are proportional to the square root of the MCC for the location at each time point. The colour gradient indicates the relative ages of the transitions (older–recent). The phylogeographic inferences of the HA and NA segments were analysed and visualized with the spatial phylogenetic reconstruction of evolutionary dynamics using data-driven documents (SpreaD3) v0.9.6^59^.

In this study, the dispersal histories of the HA and NA segments among countries and regions were inferred with a discrete phylogeographic method using BEAST, and the results are shown in Fig. 4. The 2.3.4.4 H5 HA segment originated in Guangdong Province in July 2008 (A/duck/Guangdong/wy19/2008(H5N5)-like virus), and then spread northeast, arriving in Jiangsu Province in eastern China in June 2010. Jiangsu Province then acted as a diffusion centre for multiple lines of transmission northwards (Shandong, Hebei, and Beijing) and southwards (Zhejiang). In June 2013, the H5 viruses began to spread westward to Vietnam, Laos and Sichuan province. The viruses isolated from migratory waterfowl in this study also originated from Jiangsu Province in early 2014. Similarly, the diffusion of the NA segment also began from Guangdong Province in October 2003, but travelled through a more complex migration network. Before October 2007, the viruses were restricted to Guangdong, Fujian, and Guangxi Provinces. The virus then spread northward and arrived in Zhejiang Province in February 2008. In the next several years, the viruses migrated repeatedly across eastern and southern China. These results indicate that the H5N6 viruses isolated from migratory waterfowl in this study were derived from Jiangsu Province in Eastern China.

### Evolutionary rates of the individual segments of migratory waterfowl H5N6 viruses

The phylogenetic analyses also provided the evolutionary rates of the eight gene segments from H5N6 viruses. These rates are: PB2 4.6685E-3[4.771E-3, 5.1901E-3], PB1 3.9136E-3[3.4233E-3, 4.4195E-3], PA 4.0255E-3[3.5847E-3, 4.4685E-3], HA 4.3552E-3 [3.6127E-3, 5.1492E-3], NP 3.574E-3[2.9994E-3, 4.1851E-3], NA 5.024E-3[4.4485E-3, 5.608E-3], M 3.5258E-3[2.8702E-3, 4.2164E-3] and NS 3.9349E-3[3.2275E-3, 4.6611E-3]. Evolutionary rate units are substitutions/site/year and the values in square brackets are the 95% highest posterior density (HPD) intervals. These results show that the PB2, HA and NA segments evolved slightly faster than the other segments.

According to the branch model reports of codeml program from the PAML software, the overall dN/dS ratio for the small HA dataset was 0.2312, whereas the dN/dS ratios for the two H5N6 branches were 0.1141 and 0.1294. The overall dN/dS ratio for the small NA dataset was 0.2052, whereas the dN/dS ratios for the two H5N6 branches were 0.2919 and 0.2209. These results suggest that both the HA and NA sequences were under purifying selection. Moreover, significant (probability > 95%) positively selected sites were not detected in the Bayes empirical Bayes (BEB) analysis of the site model reports. However, there were seven sites (123, 124, 140, 178, 189, 195and 266) in mature HA and two sites (83 and 143) in complete NA proteins report that had a probability of greater than 50%. These sites may be candidates for positive selection during future evolution of the viruses.

### Receptor-binding properties of migratory waterfowl H5N6 viruses

**Figure 5:**
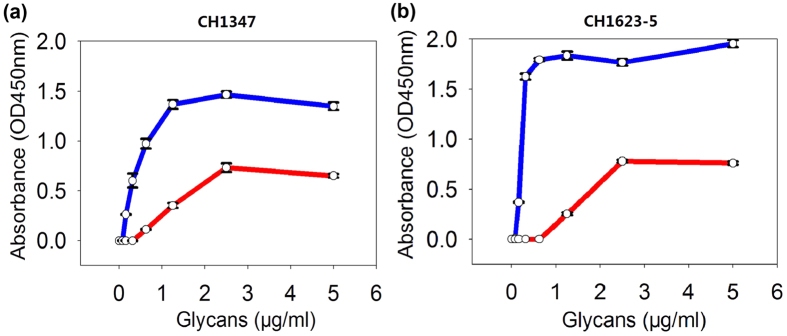
Binding of the virus to α-2,3-linked (3′SLNLN) or α-2,6-linked (6′SLNLN) sialylglycan receptors was determined with solid-phase binding assays. (**a**) CH1347 (A/Migratory waterfowl/Hubei/Chenhu1347/2014) virus; (**b**) CH1623-5 (A/Anas crecca/Hubei/Chenhu1623-5/2014) virus. Blue, binding to 3′SLNLN; red, binding to 6′SLNLN.

A genetic signature analysis showed that the HA proteins of the three wild bird isolates contained amino acids Q226 and G228 in their receptor-binding sites (Table S3), suggesting that they prefer avian-like receptors^23^. However, a T160A mutation in the HA protein was observed (Table S3), which may enhance binding to human-like receptors^24^. To evaluate the receptor-binding properties of the three isolates, the binding of H5N6 to α-2,3-linked (Neu5Acα2-3[Galβ1-4GlcNacβ1-3]_2_β-SpNH-LC-LC-biotin, 3′SLNLN) or α-2,6-linked (Neu5Acα2-6[Galβ1-4GlcNacβ1-3]_2_β-SpNH-LC-LC-biotin, 6′SLNLN) sialylglycan receptors was determined with solid-phase binding assays. CH1347 and CH1623-5 were selected for the receptor-binding analysis, and both H5N6 isolates bound to human- and avian-like receptors, but preferentially to the avian-like receptors (Fig. 5).

### Molecular markers of migratory waterfowl H5N6 viruses

The HA cleavage site in the migratory waterfowl isolates is REKRRKR (Table S3), identical to that of A/environment/Zhenjiang/C13/2013(H5N6), which suggests that they are highly pathogenic to poultry. The migratory waterfowl viruses encode a full-length NA protein, whereas some poultry H5N6 viruses have an 11-amino-acid deletion (residues 58–68) in the stalk region of NA (Table S3), which may be associated with the viral adaptation to land poultry after their introduction from wild aquatic birds^25^,^26^,^27^. Since residues at positions 591, 627 and 701 of the PB2 are thought to be critical for the mammalian adaptation of avian influenza viruses, previous studies have shown that single Q291K, E627K or D701N mutations could increase polymerase activity, viral replication in mammalian cells and the pathogenicity of influenza viruses in the BALB/c mouse model^28^,^29^,^30^,^31^. Residues Q591, E627, and D701 in the PB2 protein of wild bird H5N6 viruses suggest that these viruses have not yet adapted to mammalian hosts. In contrast, A/Sichuan/26221/2014 (H5N6) has the D701N mutation and A/Yunnan/0127/2015 (H5N6) and A/Guangzhou/39715/2014 (H5N6) have the E627K mutation and were found to infect humans. These migratory waterfowl H5N6 isolates also have a C-terminally truncated PB1-F2 protein (amino acids 58–90), which may influence their virulence in mammals^32^. No drug-resistance-associated mutations (H274Y in NA; L26P, V27A, A30T or S31N in M2^33^,^34^) were observed, indicating that these migratory waterfowl H5N6 isolates should still be sensitive to NA and M2 inhibitors. In contrast, the human H5N6 isolate A/Yunnan/0127/2015 (H5N6) contains an Amantadine-resistance mutation (S31N) in the M2 protein.

## Discussion

The results of this study showed that three H5N6 isolates were recovered from fresh droppings of migratory birds before the first human H5N6 infection case occurred. Phylogenetic analysis indicated the viruses are highly closely related to the H5N6 viruses circulating amongst poultry in China. Our results also showed that the precursor H5 and N6 AIVs have been circulating in poultry for several years, and that the reassortment events resulting in the emergence of H5N6 may have occurred in poultry in Eastern China during 2012–2013. Based on our results, it could be speculated that the H5N6 viruses in this study did not originate from wild birds. The viruses were most likely acquired from poultry, because migratory waterfowl and domestic poultry share common feeding sites around wetlands^35^. In China, live poultry markets play a key role in amplifying and disseminating AIVs across poultry market chains and maintaining the circulation of AIVs^36^,^37^. Live poultry markets are also considered areas conducive for reassortment events resulting in the emergence of novel influenza viruses^38^,^39^. The continued circulation of H5N6 amongst poultry after the first human H5N6 infection case means that additional H5N6 introduction events from poultry to humans or migratory birds are possible, and constant surveillance will be necessary to prevent future outbreaks.

**Figure 6:**
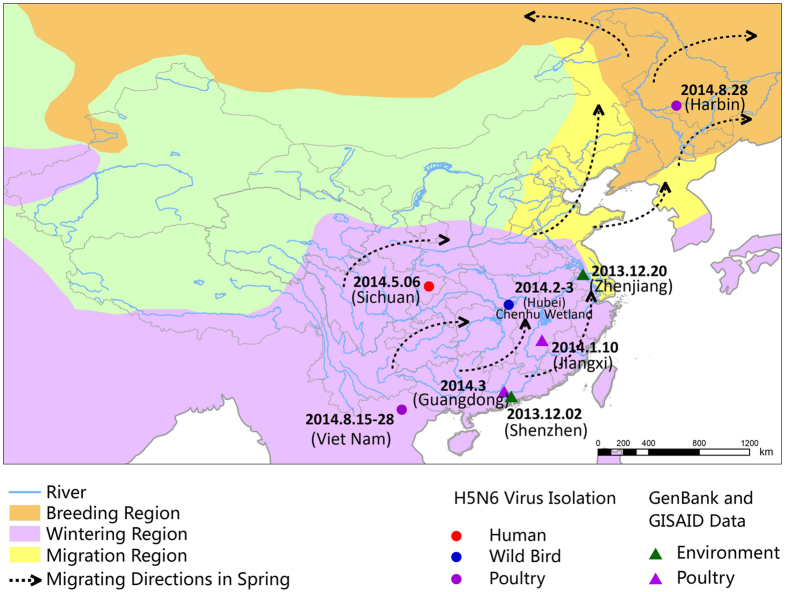
Map of the migratory direction of *Anas crecca* in spring and the sites at which the poultry H5N6 viruses were isolated. The Chenhu wetland is located in the migratory flyway. The migratory routes of *Anas crecca* in China were mapped by the ArcGIS Desktop 10.2 software (http://www.esri.com/software/arcgis/arcgis-for-desktop/).

There is a danger that H5N6 may be carried by wild birds to other areas during their long-distance migrations and then transmitted to domestic poultry. A precedent was the spread of H5N1 virus to Europe and Africa since the outbreak in Qinghai Lake after 2005^17^,^40^,^41^. Recently, the intercontinental spread of novel reassortant H5N8 viruses, from Asia to Europe and then to North America, was attributed to the migration of wild birds^42^,^43^,^44^,^45^. It has been demonstrated that the spring migration of wild ducks in Poyang Lake follows the East Asian Flyway along the coast to breeding areas in northern China, eastern Mongolia, and eastern Russia^46^. Since the Chenhu wetlands and Poyang Lake are located on the same flyway, it is possible that H5N6 may be transported to these breeding areas (Fig. 6). After H5N6 viruses were identified in migratory birds during February and March 2014, an outbreak of H5N6 in poultry was subsequently reported in north-eastern China (Heilongjiang Province) in September 2014. Although genetic information on the H5N6 strain in Heilongjiang Province is not currently available, it cannot be excluded that the H5N6 viruses may have since then disseminated to other areas along the migratory flyways.

To date, the H5N6, H5N1, H7N9, and H10N8 AIVs have caused severe infections in humans, and represent a public health threat. H5N1 viruses have caused more than 600 human infections over 17 years, whereas H7N9 viruses have caused more than 400 human infections in less than 2 years. These different outcomes may be attributed to the strong binding affinity of the H5N1 viruses for avian receptors and their weak binding affinity for human receptors, whereas H7N9 display a stronger binding affinity for human receptors compared to H5N1^47^. In this study, the receptor binding activity of the H5N6 viruses originating in migratory birds resembled that of H7N9 and H10N8 AIVs in a solid-phase binding assay^47^,^48^,^49^,^50^, and is consistent with a recent study showing that H5N6 is able to bind to receptors in the upper and lower human respiratory tract^51^. The receptor binding results can be attributed to the T160A mutation in HA, which results in the loss of a glycosylation site on the head of the HA close to the receptor binding site. This mutation was previously shown to enhance H5N1 virus binding to the α2,6-linked human receptor and playing a critical role in transmission amongst mammals^52^,^53^. In conclusion, although H5N6 does not consistently possess the mutations necessary for human infections, sustained human-to-human transmission and antiviral resistance, constant surveillance is necessary to characterize the ongoing evolution of this virus in live poultry markets to ensure that this pathogen does not become a substantial public health threat in the future.

## Materials and Methods

### Ethics statement

All studies and procedures involving animals were conducted in accordance with guidelines of animal welfare of World Organization for Animal Health. Experimental protocols were approved by the Animal Welfare and Ethical Review Committees of Wuhan Institute of Virology, Chinese Academy of Sciences.

### Sampling

The Chenhu wetlands are situated at the delta between the Yangtze River and Hanjiang River, a favourite stopover and wintering site for migratory birds in Hubei Province (Fig. 6). In the winter and spring, migratory waterfowl (mainly Anseriformes, *Phalacrocorax*, and Ardeidae) aggregate at Chenhu wetlands, making it an ideal sampling site for AIVs. Fresh and well-separated droppings of wild birds were sampled using sterile swabs in the wetland from December 2013 to March 2014. Each sample was placed in a vial containing 2 ml of viral transport medium, stored at 2–8 °C, and shipped to the laboratory within 12 h for further analysis.

### Virus isolation and sequencing

The specimens were inoculated into the allantoic cavities of 10-day-old specific-pathogen-free embryonated eggs (Beijing Merial Ltd). After incubation at 37 °C for 48–72 h, the allantoic fluid of the inoculated eggs was collected and tested for the presence of hemagglutinin. The positive samples were then confirmed for AIVs with reverse transcription–PCR. The hosts of the AIV-positive samples were identified with a mitochondrial analysis, as described previously^54^.

All gene segments were amplified with *Ex Taq* DNA polymerase (Takara) with segment-specific primers (Table S2). The PCR products were purified and sequenced with an ABI 3730 DNA Analyzer (Applied Biosystems). The data were edited and aligned with DNAMAN (version 7.0) and BioEdit (version 7.0.5.2). Whole-genome sequences of the three H5N6 influenza isolates were determined and deposited in GenBank (accession numbers: KM251462, KM251472, KM251485, KM251492, KM251502, KM251512, KM251522, KM251532, and KP083448–KP083463).

### Sequence datasets

Two different sequence datasets were generated for subsequent phylogenetic, divergence date, and phylogeographic inference analyses. Large datasets of the HA and NA segments were used for phylogenetic inference. The HA sequence dataset was composed of all available H5N6 viral HA sequences from the GenBank and GISAID databases (until May 10, 2016), the WHO H5N1 small tree, other H5N2, H5N5, and H5N8 HA sequences from the previously identified WHO H5N1 2.3.4.4 clade, and the three avian H5N6 HA sequences isolated in this study. The NA dataset was generated in a similar way. The total numbers of sequences in the HA and NA datasets were 358 and 239, respectively. Small datasets were used for the divergence date inference of all segments. The small HA and NA datasets were similar to the corresponding large datasets but without the WHO H5N1 small tree sequences. The other six datasets of the internal segments were derived from the top 250 Blastn^55^ hits against the nt database. For all eight datasets, identical sequences and sequences without definite collection dates were removed to accelerate the Bayesian inference and improve the precision of the analysis. The numbers of sequences in each segment dataset were: PB2 101, PB 185, PA 89, HA 72, NP 58, NA 124, M 128, and NS 87.

### Sequence analyses

The alignment of each dataset was generated with Clustal Omega^56^ (version 1.2.1) with 1,000 iterations, and codon alignments were applied to all small datasets. The alignment lengths for each small dataset were: PB2 2,277 nucleotides (nt), PB1 2,271 nt, PA 2,148 nt, HA 1,701 nt, NP 1,494 nt, NA 1,410 nt, M 981 nt, and NS 819 nt. The phylogenetic inferences and classification of the HA and NA segments in the large datasets were made with RAxML^57^ (version 8.2.6) under the GTRGAMMA model with 1,000 bootstrap replicates. The overall rate of evolutionary change and the time of TMRCA for all segments in the small datasets were estimated with BEAST^58^ (version 2.3.2), with the HKY85 plus Gamma nucleotide substitution model and a relaxed clock. The BEAST software was also used to infer the discrete phylogeography of the HA and NA segments in the small datasets. All chains were run in 50,000,000 generations with 10% burn-in, and the effective sample size (ESS) values in the results were greater than 200. The phylogenetic trees were visualized and annotated with FigTree (http://tree.bio.ed.ac.uk/software/figtree/). The phylogeographic inferences of the HA and NA segments were analysed and visualized with the spatial phylogenetic reconstruction of evolutionary dynamics using data-driven documents (SpreaD3) version 0.9.6^59^.

The selection pressures on the HA and NA segments at codon sites and lineages were estimated with PAML^60^. The branch model was used to estimate the selection pressures of individual lineages, and the site model was used to identify the sites of positive selection.

### Solid-phase binding assay

Receptor-binding specificity was analysed with a solid-phase direct-binding assay, as described previously^61^. Briefly, the wells of plates were coated with serial dilutions of biotinylated glycans, 3′SLNLN and 6′SLNLN, overnight at 4 °C. After the glycopolymer solution was removed, the plates were blocked, washed, and incubated in a solution containing 64 HA units of influenza virus. After the plates were washed again, chicken antisera directed against A/Anas crecca/Hubei/Chenhu1623-5/2014 (H5N6) was added to each well. The wells were then washed and incubated with horseradish-peroxidase-linked goat anti-chicken antibody (BETHYL). The plates were washed and incubated with TMB (Sigma) for 10 min at room temperature. The reaction was stopped with 0.05 ml of 0.5 M H_2_SO_4_ and the absorbance was read at 450 nm.

## Additional Information

**How to cite this article**: Bi, Y. *et al*. Novel avian influenza A (H5N6) viruses isolated in migratory waterfowl before the first human case reported in China, 2014. *Sci. Rep.* **6**, 29888; doi: 10.1038/srep29888 (2016).

## Supplementary Material

Supplementary Information
