# Supplementary material for: Novel avian influenza A (H5N6) viruses isolated in migratory waterfowl before the first human case reported in China, 2014
Source: Sci Rep. 2016 Jul 19;6:29888. doi: 10.1038/srep29888 (PMC4949417; doi:10.1038/srep29888)
Supplement: Supplementary Information [file srep29888-s1.doc]

**Supplementary Information**

**Title: Novel avian influenza A (H5N6) viruses isolated in migratory waterfowl before the first human case reported in China, 2014**

Authors: Yuhai Bi, Haizhou Liu, Chaochao Xiong, Di Liu, Weifeng Shi, Mingxin Li, Siling Liu, Jing Chen, Guang Chen, Yong Li, Guoxiang Yang, Yongsong Lei, Yanping Xiong, Fumin Lei, Hanzhong Wang, Quanjiao Chen, Jianjun Chen and George F Gao

Table S1. The information of sampling and isolation

| Sampling Date | Source Type | Sample no. | NDV positive | AIV positive | H5N6 positive |
| --- | --- | --- | --- | --- | --- |
| 2013.11.22 | Feces | 104 | 0 | 0 | 0 |
| 2013.12.16 | Feces | 280 | 2 | 2 | 0 |
| 2014.01.17 | Feces | 377 | 9 | 2 | 0 |
| 2014.02.26 | Feces | 360 | 3 | 3 | 2 |
| 2014.03.20 | Feces | 119 | 3 | 1 | 1 |
| Total | - | 1240 | 17 | 8 | 3 |

Table S2. The primer sets for AIV detection and segments PCR

| Target gene* | Primer name | Primer Sequence |
| --- | --- | --- |
| M fragment | M229L | 5′-AAgCgTCTACgCTgCAgTCC-3′ |
|  | M229U | 5′-TTCTAACCgAggTCgAAAC-3′ |
| PB2 | Ba-PB2-1F | 5'-TAggAgCgAAAgCAggTC-3' |
|  | PB2-1-1205R | 5’-TCYTCYTgTgARAAYACCAT-3’ |
|  | PB2-2-1105F | 5’-TAYgARgARTTCACAATggT-3’ |
|  | Ba-PB2-2341R | 5'-ggAgTAgAAACAAggTCgTTT-3' |
| PB1 | Bm-PB1-1F | 5'-ATTTAgCgAAAgCAggCA-3' |
|  | PB1-1-1262R | 5’-TTRAACATgCCCATCATCAT-3’ |
|  | PB1-2-1142F | 5’-ARATACCNgCAgARATgCT-3’ |
|  | Bm-PB1-2341R | 5'-TggAgTAgAAACAAggCATTT-3' |
| PA | Bm-PA-1F | 5'-gggAgCgAAAgCAggTAC-3' |
|  | PA-1-1498R | 5’-TNgTYCTRCAYTTgCTTATCAT-3’ |
|  | PA-2-747F | 5’-CATTgAgggCAAgCTTTC-3’ |
|  | Bm-PA-2233R | 5'-CCggAgTAgAAACAAggTACTT-3' |
| NP | Bm-NP-1F | 5’-TAggAgCAAAAgCAgggTA-3’ |
|  | Bm-NP-1565R | 5’-TggAgTAgAAACAAgggTATTTTT-3’ |
| NA | Ba-NA-1F | 5’-ATggAgCAAAAgCAggAgT-3’ |
|  | Ba-NA-1413R | 5’-ggCCAgTAgAAACAAggAgTTTTTT-3’ |
| MP | M-Bm-1F | 5’-ACggAgCAAAAgCAggTAg-3’ |
|  | M-Bm-1027-R | 5’-CggAgTAgAAACAAggTAgTTTTT-3’ |
| NS | Bm-NS-1F | 5’-TggAAgCAAAAgCAgggTg-3’ |
|  | Bm-NS-890R | 5’-TggAgTAgAAACAAgggTgTTTT-3’ |
| *PB2, basic polymerase 2; PB1, basic polymerase 1; PA, acidic polymerase; HA, hemagglutinin; NP, nucleoprotein; NA, neuraminidase; MP, matrix protein; NS, nonstructural protein. | | |

Table S3**.** Molecular characterizations of H5N6 viruses

| Viruses (H5N6) ^†^ | Collection  date | HA (H3 numbering) | | | | | |  | PB2 | | | | NA | | | |  | | M2 | | | | |  | | PB1-F2 | |  |
| --- | --- | --- | --- | --- | --- | --- | --- | --- | --- | --- | --- | --- | --- | --- | --- | --- | --- | --- | --- | --- | --- | --- | --- | --- | --- | --- | --- | --- |
|  |  | Connecting  peptide | 160 | 224 | 226 | 228 | 318 |  | | 591 | 627 | 701 | |  | 274* | Stalk deletion | |  | | 26 | 27 | 30 | 31 | |  | | 58-90 truncated | |
| CH1306 | 2014-2-20 | REKRRKR | A | N | Q | G | T |  | | Q | E | D | |  | H | No | |  | | L | V | A | S | |  | | Yes | |
| CH1347 | 2014-2-20 | REKRRKR | A | N | Q | G | T |  | | Q | E | D | |  | H | No | |  | | L | V | A | S | |  | | Yes | |
| CH1623-5 | 2014-3-20 | REKRRKR | A | N | Q | G | T |  | | Q | E | D | |  | H | No | |  | | L | V | A | S | |  | | Yes | |
| SC26221 | 2014-4-21 | REKRRKR | A | N | Q | G | T |  | | Q | E | N | |  | H | No | |  | | L | V | A | S | |  | | Yes | |
| YN0127 | 2015-2-6 | RERRRKR | A | N | Q | G | T |  | | Q | K | D | |  | H | 58-68 | |  | | L | V | A | N | |  | | No | |
| GZ39715 | 2014-12-11 | RERRRKR | T | N | Q | G | T |  | | Q | K | D | |  | H | 58-68 | |  | | L | V | A | S | |  | | No | |
| ZJC13 | 2013-12-20 | REKRRKR | A | N | Q | G | T |  | | Q | E | D | |  | H | No | |  | | L | V | A | S | |  | | Yes | |
| GD01 | 2014-03 | RERRRKR | A | N | Q | G | T |  | | Q | E | D | |  | H | 58-68 | |  | | L | V | A | S | |  | | Yes | |
| SZ25-24 | 2013-12-02 | RERRRKR | A | N | Q | G | T |  | | Q | E | D | |  | H | 58-68 | |  | | L | V | A | S | |  | | Yes | |
| JX95 | 2014-01-10 | RERRRKR | A | N | Q | G | T |  | | Q | E | D | |  | H | 58-68 | |  | | L | V | A | S | |  | | Yes | |

*N2 numbering.

^†^CH1306, A/Migratory Waterfowl/Hubei/Chenhu1306/2014; CH1347, A/ Migratory Waterfowl/Hubei/Chenhu1347/2014; CH1623-5, A/Anas crecca/Hubei/Chenhu1623-5/2014; SC26221, A/Sichuan/26221/2014; YN0127, A/Yunnan/0127/2015; GZ39715, A/Guangzhou/39715/2014; ZJC132, A/environment/Zhenjiang/C13/2013; GD01, A/duck/Guangdong/GD01/2014; SZ25-24, A/environment/Shenzhen/25-24/2013; JX95, A/duck/Jiangxi/95/2014.
